# Co-production of two whole-school sexual health interventions for English secondary schools: positive choices and project respect

**DOI:** 10.1186/s40814-020-00752-5

**Published:** 2021-02-17

**Authors:** Ruth Ponsford, Rebecca Meiksin, Sara Bragg, Joanna Crichton, Lucy Emmerson, Tara Tancred, Nerissa Tilouche, Gemma Morgan, Pete Gee, Honor Young, Alison Hadley, Rona Campbell, Chris Bonell

**Affiliations:** 1 Department of Public Health, Environments & Society, London School of Hygiene and Tropical Medicine, 15-17 Tavistock Place, London, WC1H 9SH UK; 2 Centre for Sociology of Education and Equity, UCL Institute of Education, 20 Bedford Way, London, WC1H 0AL UK; 3 Population Health Sciences, Bristol Medical School, University of Bristol, 39 Whatley Road, Bristol, BS8 2PS UK; 4 Sex Education Forum, National Children’s Bureau, 23 Mentmore Terrace, London, E8 3PN UK; 5 Centre for Maternal and Newborn Health, Liverpool School of Tropical Medicine, Pembroke Place, Liverpool, L3 5QA UK; 6 School of Social Sciences, Cardiff University, 1-3 Museum Place, Cardiff, CF10 3BD UK; 7 Teenage Pregnancy Knowledge Exchange, University of Bedfordshire, University Square, Luton, LU1 3JU UK

**Keywords:** Co-production, Complex health interventions, Evaluation, Teenage pregnancy, Sexual health, Dating and relationships violence, Sexual harassment, Public health, Relationships and sex education

## Abstract

**Background:**

Whole-school interventions represent promising approaches to promoting adolescent sexual health, but they have not been rigorously trialled in the UK and it is unclear if such interventions are feasible for delivery in English secondary schools. The importance of involving intended beneficiaries, implementers and other key stakeholders in the co-production of such complex interventions prior to costly implementation and evaluation studies is widely recognised. However, practical accounts of such processes remain scarce. We report on co-production with specialist providers, students, school staff, and other practice and policy professionals of two new whole-school sexual heath interventions for implementation in English secondary schools.

**Methods:**

Formative qualitative inquiry involving 75 students aged 13–15 and 23 school staff. A group of young people trained to advise on public health research were consulted on three occasions. Twenty-three practitioners and policy-makers shared their views at a stakeholder event. Detailed written summaries of workshops and events were prepared and key themes identified to inform the design of each intervention.

**Results:**

Data confirmed acceptability of addressing unintended teenage pregnancy, sexual health and dating and relationships violence via multi-component whole-school interventions and of curriculum delivery by teachers (providing appropriate teacher selection). The need to enable flexibility for the timetabling of lessons and mode of parent communication; ensure content reflected the reality of young people’s lives; and develop prescriptive teaching materials and robust school engagement strategies to reflect shrinking capacity for schools to implement public-health interventions were also highlighted and informed intervention refinements**.** Our research further points to some of the challenges and tensions involved in co-production where stakeholder capacity may be limited or their input may conflict with the logic of interventions or what is practicable within the constraints of a trial.

**Conclusions:**

Multi-component, whole-school approaches to addressing sexual health that involve teacher delivered curriculum may be feasible for implementation in English secondary schools. They must be adaptable to individual school settings; involve careful teacher selection; limit additional burden on staff; and accurately reflect the realities of young people’s lives. Co-production can reduce research waste and may be particularly useful for developing complex interventions, like whole-school sexual health interventions, that must be adaptable to varying institutional contexts and address needs that change rapidly. When co-producing, potential limitations in relation to the representativeness of participants, the ‘depth’ of engagement necessary as well as the burden on participants and how they will be recompensed must be carefully considered. Having well-defined, transparent procedures for incorporating stakeholder input from the outset are also essential. Formal feasibility testing of both co-produced interventions in English secondary schools via cluster RCT is warranted.

**Trial registration:**

Project Respect: ISRCTN12524938. Positive Choices: ISRCTN65324176

## Key messages regarding feasibility

- Systematic reviews suggest that whole-school interventions are promising approaches to addressing adolescent sexual health, but it is unclear if delivery in English secondary schools is feasible. It is widely recognised that such complex interventions must be carefully developed with intended recipients, implementers and other relevant stakeholders to maximise their contextual applicability prior to formal pilot and feasibility studies.
- Based on formative qualitative inquiry with school staff, students and other youth and policy stakeholders, our findings suggest that multi-component, whole school interventions employing teacher delivered curriculum to address unintended teenage pregnancy and dating and relationships violence (DRV) may be appropriate and feasible for delivery in English secondary schools providing they are adaptable to individual school settings; involve careful teacher selection; limit additional burden on staff; and accurately reflect the realities of young people’s lives.
- Co-production activities informed important refinements to the design of Positive Choices and Project Respect that are likely improve their applicability and quality of implementation in English secondary schools. Following these refinements, formal feasibility testing of both interventions via pilot cluster randomised trial is warranted.

## Background

Despite significant declines in recent decades, the teenage birth rate in the UK remains higher than in other comparable western European countries and reduction by region varies considerably [1–3]. Teenagers are also the most likely group to experience unintended pregnancy with around half of conceptions to under 18 s in England and Wales ending in abortion, this increasing to over 60% among those under 16 [2]. Diagnosis of STIs (sexually transmitted infections) in England remains highest among those aged 15-24 [4], while non-volitional sex (NVS) and dating and relationships violence (DRV) in the teenage years are widely, and likely also under, reported in the UK [5–7]. The costs of unintended pregnancy, STIs and domestic violence to health and public services are significant [8, 9]. Preventing unintended teenage pregnancy and improving sexual health among young people in England, therefore, remains a priority.

There is good evidence that school-based relationships and sex education (RSE) is a key element in preventing unintended pregnancy and promoting sexual health [10–14]. Interventions involving whole-school in addition to classroom elements represent particularly promising approaches over basic curriculum only programmes, which systematic reviews suggest often have limited and inconsistent impact on behavioural outcomes [11, 13, 15–18]. Whole-school action can include changes to school policies and practice to support promotion of sexual health; student participation in planning and delivering activities; school-wide health promotion campaigns; parent engagement; and improving student access to contraceptive, sexual health and other relevant support services. Recent reviews suggest that interventions involving whole school elements can have significant and sustained impacts on delaying sexual debut [19]; and increasing contraception use and reducing pregnancy rates [20]. Evidence also suggests that interventions involving whole-school actions can have long-term impact on victimisation and perpetration of sexual and physical violence [21, 22]. Whole school approaches to addressing unintended teenage pregnancy and sexual health, however, have not been rigorously tested in the UK and it is unclear if such interventions are feasible for delivery in English secondary schools.

Prior to undertaking formal pilot and feasibility studies, the need for proper investment and rigour in the development of complex interventions, like whole school interventions, is increasingly recognised. As such, a number of frameworks have emerged to support the development of complex interventions like these [23–33]. Most propose a phased and iterative approach involving identification of similar effective interventions, their component parts and/or mechanisms of action in the existing literature, developing intervention theory, and prototyping and testing delivery models and materials. The importance of stakeholder involvement across phases is emphasised, with potential beneficiaries and intervention providers viewed as having unique insights into how health problems are constructed and maintained, and the local context in which interventions will be delivered [34]. Stakeholders are thus recognised as having a valuable contribution to make as ‘co-producers’ of interventions by, for example, identifying appropriate and relevant intervention aims and content; contributing to the delineation of theories of change; highlighting facilitators and barriers to implementation and acceptability; and identifying potential unintended consequences and ways of addressing these [27, 28, 34, 35].

This increasing interest in co-production in intervention design reflects broader trends towards greater involvement of policy-makers, practitioners and the wider public in research, motivated by a range of concerns from democratising and improving the transparency of research, to enhancing relevance, quality and uptake in policy and practice, as well as longstanding interests in patient participation in healthcare improvement where service users are seen as best placed to advise on service deisgn and delivery [36–42].

With regard to children and young people specifically, their fundamental human right to participate in decisions and actions that affect them, including the design of programmes and policies aiming to serve them, is enshrined in the UN Convention on the Rights of the Child [43]. Sex educators too have for long advocated for the involvement of young people as co-producers in the development of RSE materials to ensure that these keep pace with the constantly shifting social and technological landscape in which young people experience and conduct their lives and relationships [44–47].

There is, however, some contention over what ‘counts’ as co-production in practice [48]. While in its initial intended sense co-production implies a level of collaboration and parity of power between researcher and co-producer, in intervention design the term has come to describe a diverse set of goals and activities ranging from stakeholders merely being informed or consulted, through to them having the authority and control to make decisions and shape the content and direction of interventions [41, 49, 50].

Yet, despite increased interest in co-production in the development of complex interventions, practical accounts of such processes remain scarce[51]. Such accounts are critical for furthering understanding of the role and value of co-production in intervention design and for informing practical strategies for carrying out such work. In this paper, we report our approach to the co-production of two multi-component, whole-school sexual health interventions for implementation in English secondary schools: ‘Positive Choices’ aimed at preventing unintended teenage pregnancy and improving sexual health and ‘Project Respect’ aimed at addressing DRV and sexual harassment in schools. We describe how the involvement of potential recipients (students), implementers (school staff) and wider youth and practitioner and policy stakeholders informed and improved the design of these two interventions prior to formal feasibility testing via cluster randomised control trial (RCT). We also reflect on some of the challenges and tensions involved in the process of coproduction and the extent to which we can claim to have involved stakeholders as collaborators in our research. Our findings provide valuable insights for those planning the design and delivery of similar health interventions in secondary schools in England and for those considering similar co-production activities with students, school staff and other stakeholders.

## Methods

### Initial intervention design

Positive Choices and Project Respect were both designed as new evidence-based interventions, rather than as replications of existing ones. Design began by defining primary and secondary outcomes, a theory of change and set of components for each intervention based on existing evidence.

Positive Choices aimed to reduce unintended teenage pregnancy (primary outcome). Secondary outcomes included delayed sexual debut, reduced numbers of sexual partners, increased use of contraception and improved educational attainment. Planned intervention components comprised a report for schools on student sexual health and RSE needs informed by student surveys; a School Health Promotion Council (SHPC) involving at least six staff and six students to coordinate intervention activities and tailor the intervention to local needs; a teacher-delivered classroom curriculum for year 9 students (aged 13–14); parent newsletters and homework; student-led social marketing campaigns; and a SHPC-led review of school and local sexual-health services. Training and a manual were included for staff facilitating the council, curriculum and campaigns.

Project Respect’s primary outcome was to prevent dating and relationships violence (DRV). Secondary outcomes included reduced sexual harassment, unintended pregnancy and sexually transmitted infections, delayed sexual debut, reduced numbers of sexual partners, and improved use of contraception, psychological functioning and educational attainment. The planned intervention comprised the following: a manual and training for key staff to coordinate intervention activities; training by these staff for other staff on preventing DRV; staff and student mapping of ‘hotspots’ for DRV on site and revision of staff patrols to address these; review of school policies to address DRV; a teacher-delivered classroom curriculum for year 9/10 students (aged 13–15); providing students with the ‘Circle of 6’ app for seeking support when experiencing or at risk of DRV; and parent information about DRV.

Initial design of both interventions was informed by studies of previous interventions reported as effective in promoting various sexual health outcomes relevant to the prevention of unintended teenage pregnancy and DRV in randomised trials from the US and Australia [22, 52–60].

**Fig. 1:**
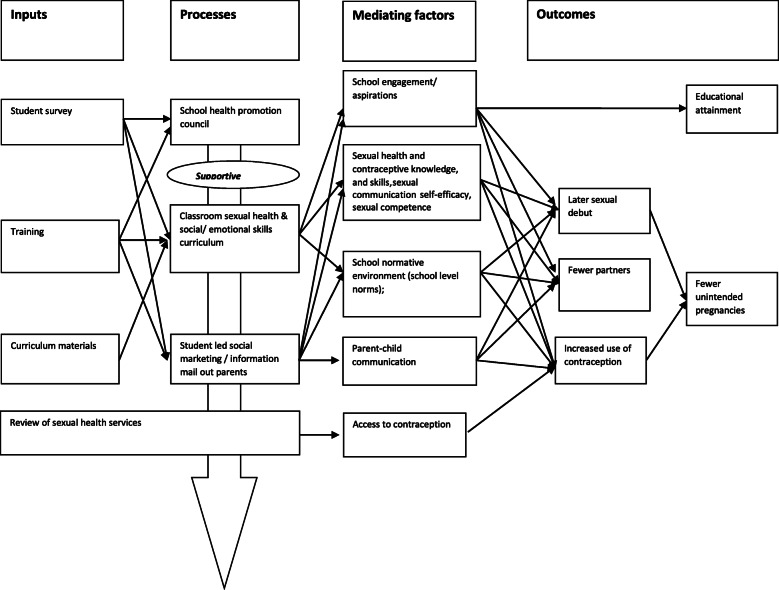
Positive Choices logic model

Positive Choice’s theory of change (Fig. 1) was informed by social marketing theory [61, 62] [63], models of school change [64], social influence theory [65] and social cognitive theory [66], and focused on achieving positive sexual health outcomes by improving contraceptive and safer sex knowledge and skills; self-efficacy to communicate about sex [67]; sexual competence [68]; communication at home about relationships and sex; and school-wide social norms supporting positive relationships/sexual health. Student participatory elements were also theorised to promote connection to school (a protective factor for sexual risk taking [69, 70]) and improve academic attainment. Although the main outcome measure was unintended teenage pregnancy, the intervention, therefore, took a broader approach to addressing sexual health focussing on a range of related intermediate outcomes.

**Fig. 2:**
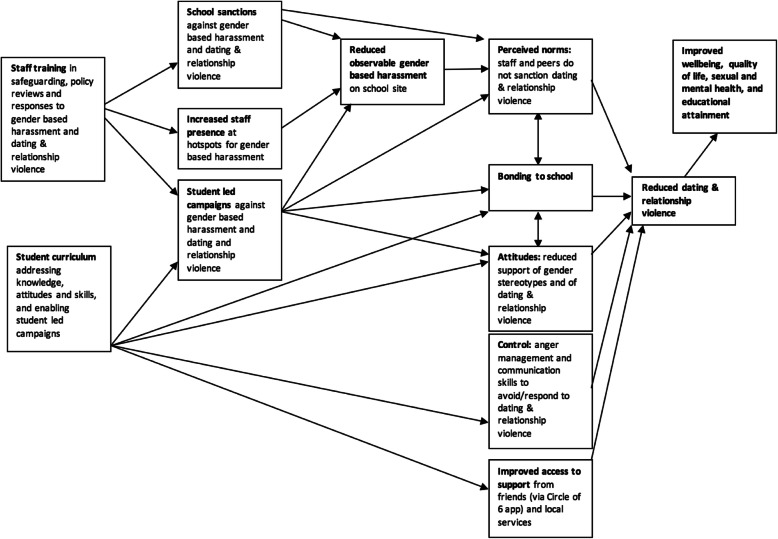
Project Respect logic model

Project Respect’s theory of change (Fig. 2) was underpinned by the theory of planned behaviour [71] and the social development model [72], which informed a focus on challenging student attitudes and perceived social norms about gender, appropriate behaviour in relationships and violence, and promoting sense of control over behaviour. This approach was also supported by reviews which suggest that DRV prevention should both challenge attitudes and perceived norms concerning gender stereotypes and violence, and support the development of skills and control over behaviour [73].

The initial design of both interventions was thus primarily informed by academic theory and research. However, the drafting of the funding proposals for each study also involved preliminary consultation with a staff member from five different schools involved in a schools research network led by the research team and with young people from ALPHA (Advice Leading to Public Health Action): a young people’s research advisory group led by the Centre for the Development and Evaluation of Complex Interventions for Public Health Improvement (DECIPHer) at Cardiff University. The group comprises young people trained in public health and related research methods who work with researchers and policy-makers to provide insights on study design and policy initiatives from a youth perspective.

These consultations informed our decision to focus the curriculum on year 9 and 10 students; suggested that students and staff were supportive of intervention components and the whole-school approach to address unintended teenage pregnancy, sexual health and DRV; and that although some components were already being delivered in some schools, none were using a coherent whole-school programme to address these outcomes.

### Funded intervention elaboration

#### Overview

Following initial design, research funding was obtained for ‘optimisation’ and piloting of each intervention prior to formal feasibility testing. In this case, optimisation involved the further specification and development of the intervention components led by researchers in collaboration with specialist agencies who were to provide each intervention and involved consultation with secondary school staff and students; and other youth and policy stakeholders to produce fully elaborated interventions with materials appropriate for English secondary schools.

The Sex Education Forum (SEF) was the specialist development partner and provider for Positive Choices and the National Society for the Prevention of Cruelty to Children (NSPCC) for Project Respect. Part of the National Children’s Bureau (NCB) charity, SEF advocates and provides resources for delivery of quality RSE in England. The NSPCC is also a charity, focused on preventing child abuse.

Optimisation involved a review by researchers and SEF/NSPCC of evaluation reports and, where available, intervention materials from the interventions that informed Positive Choices and Project Respect; initial consultation with staff and students from secondary schools in England on intervention content, delivery and materials; drafting by SEF/NSPCC of intervention materials in collaboration with research staff; further consultation with schools, other young people (ALPHA) and policy stakeholders on intervention format and materials; and intervention refinement prior to piloting.

#### Consultation with schools

For Positive Choices, initial consultation with students and staff holding a range of roles in one London secondary school was carried out in June 2017 prior to the development of intervention delivery models and materials, which were to be piloted for feasibility and acceptability in the same school from September 2017. The session involved teachers and students from year 8 and focused on acceptability of intervention aims, components, content and proposed modes of delivery; preferences for the content and format of the student needs report and the manual guiding the intervention; and identifying any perceived challenges to implementation. Following a presentation on intervention aims and components given by a member of the SEF intervention provider team, students and staff were split to discuss their perspectives on the intervention. The staff group was facilitated by a researcher (RP) while the student group was facilitated by the SEF representative. Coloured cards with details of each of the intervention components on were also used to help prompt discussion around acceptability and feasibility in each of the groups. Staff were provided with sample materials from a draft needs report and manual to prompt further discussion around the format of guidance materials. Focussing specifically on the curriculum topics, year 8 students were asked to discuss what topics they had previously learnt about in RSE and then to write down on post-it notes something they would like to learn more about in year 9. Students were then asked to review the topics the intervention developers had signalled for inclusion in the curriculum and see which of theirs were included and which were missing. In the case of Positive Choices, further planned consultation on intervention materials once developed was not possible due to limited capacity for participation from the school.

For Project Respect, consultation involved two sets of workshops at four schools (two in south-east and two in south-west England). The first set of workshops was conducted in three of the schools in May 2017 and involved a mix of staff and students. These focused on acceptability of intervention aims, components, delivery models and the format of the intervention including staff training, the manual and the curriculum as well as wider issues of implementation. As with the Positive Choices workshop, the intervention provider (NSPCC) gave a presentation detailing the intervention aims and components. At various points, the intervention provider paused the presentation to discuss the content of the slides and get direct feedback on the elements that had just previously been presented. A set of prompt questions were predefined to explore participants’ perspectives around relevance and acceptability of intervention aims and approaches, and feasibility of implementation. Students and staff were separated for at least part of the discussion. This data was also supplemented by a telephone interview with a staff-member at the fourth school where it was not possible to arrange to visit.

The second sessions for Project Respect occurred in July 2017, involving staff and students in consultations in three schools. These explored appropriate terminology for relationships and abusive behaviours; sought feedback on draft curriculum materials and suitability for delivery in English schools; and considered the role of social media in the conduct of young people’s relationships and DRV. In these sessions, following an introductory presentation given by the intervention provider and an ice breaker activity, participants were divided into three separate discussion groups for staff, year 9 and year 10 students. Students were asked to brainstorm the terminology they used to describe sexual and romantic relationships; DRV; and sexual harassment. They were then asked to discuss the role social media played in their dating and intimate relationships. Students were also provided with lesson plans and slides for several lessons and asked to discuss their impressions of them. A set of prompts was devised to elicit responses around the relevance of content and acceptability of pedagogical approaches. Staff were asked about what made curriculum materials most useful and what would make the PR lesson plans easy to use. They were asked to give feedback on handouts of draft materials for one specific lesson (lesson plan, slides and student handouts); how staff would prepare for lessons; how teaching staff would likely be selected and their perspective on the use of external educators. They were also asked about the role of social media in young peoples’ dating and relationships and in DRV. In each Project Respect workshop, discussion was facilitated by the NCPCC representative and at least two researchers (JC, GM, RM, NT, TT).

The Positive Choices and the second wave of the Project Respect sessions were audio-recorded. Field notes were also taken during or directly after all sessions. Based on these recordings and field notes, summary reports for each workshop were prepared. In terms of recruitment, schools were asked to select a range of teaching and support staff with involvement in RSE or Personal Social Health Economic (PSHE) education and a diverse group of students broadly representative of the student population in year 8 for Positive Choices and in years 9 and 10 for Project Respect.

#### Consultation with ALPHA group

For Positive Choices, two workshops were held with the ALPHA group in July 2017 and April 2018, to explore young people’s perspectives on parent engagement and the acceptability and potential challenges of implementing student-led social marketing campaigns in schools. For Project Respect, the ALPHA group were consulted on draft lesson plans in October 2017. All ALPHA workshops involved interactive group-based discussion activities employing prompt material from each of the interventions. All activities were designed by the groups’ professional facilitator (PG) and approved by researchers. All ALPHA workshops were facilitated, audio-recorded and summaries of the discussions drafted by the group’s facilitator.

#### Consultation with practitioners and policy-makers

In March 2018, we convened a meeting of sexual health and RSE practitioners and policy-makers from governmental and non-governmental organisations to discuss the Positive Choices and Project Respect projects jointly. Participants were identified by the research team and invited by email to join a stakeholder group to advise on intervention and research design. Following presentations on each intervention, participants provided feedback via small-group discussion on questions specified by researchers, focusing specifically on the acceptability of intervention design and feasibility of  implementation. Drawing on facilitator notes, researchers drafted a summary of the event that was also shared with participants.

#### Ethics

Ethics approval for co-production procedures was granted by the London School of Hygiene and Tropical Medicine research ethics committee on 25th January 2017 for Project Respect and 5th June 2017 for Positive Choices. Students and staff were treated as research participants and provided with written information about the research 1 week beforehand, as well as verbally just prior to the research. Participants were informed that they could stop taking part at any time or choose not to answer any questions. All completed written opt-in consent/assent forms. Parents of participating students were provided with information and could opt their children out by contacting the research team or their child's school.

ALPHA participants gave written consent for their participation as research advisors on DECIPHer affiliated studies and for their contributions to be shared anonymously for all general purposes in relation to DECIPHer’s work. Consultation with practitioners and policy-makers was treated as public engagement rather than research, so specific ethical review and consent were not sought. Participants were made aware of how their contributions would be used and received a summary of discussion, to which they could suggest amendments.

#### Incorporation of findings from consultation into intervention design

The summaries prepared for each of the above activities were shared with the specialist provider agencies for each intervention. Providers and researchers discussed the summaries arriving at a negotiated consensus about how these should inform content, models of delivery and the format of materials.

## Results

In the following sections, we report the findings from consultations with school staff, students and other youth and policy stakeholders, and describe how these informed the design of both interventions. These are also summarised in Table 1.

**Table 1:** Table of how stakeholder feedback informed intervention design

Intervention	Stakeholder feedback	Stakeholder group	How incorporated into intervention design
**PR and PR**	Intervention aims appropriate and relevant.	Consultation with students, teachers, ALPHA and policy stakeholders	Confirmed planned approaches
**PR and PR**	Interventions components appropriate. Tailoring to student needs particularly valued.	Consultation with students and teachers	Supported planned approaches
**PR**	Concern over student preference informing selection of whole curriculum.	Consultation with teachers	Curriculum developed with essential and ‘add on’ lessons the selection of which was to be informed by the student needs assessment.
**PR**	Train-the-trainer model acceptable and helpful in reducing number of teachers needing to be released for whole day training.	Consultation with teachers	Confirmed planned approaches
**PR**	Curriculum lessons need to be adaptable for split delivery over shorter than an hour slots.	Consultation with teachers	Built in to design of curriculum lessons for both PC and PR
**PR and PC**	Manual materials need to be concise and to the point. Supporting evidence and theory should be provided as appendices.	Consultation with teachers	Manual materials for both projects developed with these points in mind.
**PR and PC**	Curriculum materials should be ‘plug and play’ so staff with limited confidence, experience or time could deliver an effective lesson.	Consultation with teachers	It was agreed that pragmatically and to ensure fidelity of implementation prescriptive materials should be developed for both interventions.
**PR and PC**	Materials should be adaptable for more experienced or confident teachers	Consultation with teachers	Essential material and where adaption was possible was highlighted in both interventions and a selection of additional materials and options for differentiation included.
**PR and PC**	Options to adapt lesson content to schools’ existing provision	Consultation with teachers/Professional and policy stakeholder event	Assessed on a case by case basis following a review of what schools have already covered and materials used.
**PR and PC**	Intervention materials should be provided in electronic format and in hard copy.	Consultation with teachers	Materials supplied electronically to all staff and in online format for PC. Hard copies handed out at trainings.
**PR and PC**	Introduction of interventions at an earlier stage in years seven when students are aged 11-12 or eight when students are aged 12-13.	Consultation with students	Contradicted teacher and student feedback in earlier consultation. Was agreed with specialist provider agencies that intervention content was appropriate for years 9 for PC and 9 and 10 for PR.
**PR**	Small group, discussion activities and ‘real life scenarios to reflect on appreciated by young people.	Consultation with students and ALPHA	Confirmed planned approaches on PR and PC.
**PR**	Subtler or less obvious forms of abuse should be covered by the intervention	Consultation with students	Confirmed planned approaches in PR.
**PR**	Appropriate signposting and support should be provided for students, including how to support friends who disclose abuse.	Consultation with ALPHA	Built in to each lesson for both interventions.
**PR**	Materials should accurately reflect the lives of young people including the role of social media in DRV and online technologies in the conduct of young people's lives and relationships.	Consultation with students and ALPHA	Informed curriculum content
**PR**	Young people use a range of terms to define dating and relationships	Consultation with students	Terms and meanings used in the intervention defined clearly for both students and staff in intervention materials.
**PC and PR**	Teacher educators can be acceptable and valued, but careful selection of teachers is required.	Consultation with students	Confirmed planned approaches, but schools were encouraged to select trained teaching staff and those with an interest and commitment to teaching these topics.
**PC and PR**	External educators may increase sense of student safety in the classroom and bring specialist, expert knowledge to lessons.	Consultation with staff and students	Model promotes training staff to be competent in teaching topics covered by each of the interventions. Budget did not allow for the inclusion of external experts to deliver lessons for each school, although schools were able to source these as part of their usual provision if they so wished.
**PC and PR**	Some ‘sensitive’ topics should be taught in single sex lessons.	Consultation with staff and students	Generally, runs against best practice for the delivery of RSE. Guidance was provided for schools that lessons should be taught in mixed sex groups to enable the sharing of ideas and discussion across genders, and model real life experiences. Also, potential alienation of trans, non-binary or questioning students.
**PC**	Student led social marketing campaigns needs some wider oversight to ensure student messaging is consistent with programme aims	Consultation with ALPHA	Oversight to be provided by the School Health Promotion Council (SHPC). Specific links and responsibilities for SHPC oversight built in to design of student led social marketing component.
**PR and PC**	Flexibility in the mode of parent engagement. Parent engagement materials should be sensitive to local home cultures.	Consultation with staff, students and ALPHA.	Mode of engaging with parents (e.g. for disseminating information and newsletters) and exact content of information left open for schools.
**PC**	Homework could breach parent/child boundaries	Consultation with ALPHA	In line with SEF intended plan, homework assignments remain defined as an essential part of the curriculum, but introduced carefully.
**PR and PC**	Deep engagement with senior leadership members at participating schools to encourage school commitment	Professional and policy stakeholder event	For PC face to face meetings organised with all head teachers
**PR and PC**	Disseminate information about interventions throughout the school community to awareness throughout the school and promote school commitment	Professional and policy stakeholder event	For PC guidance on launch activities and disseminating information provided in intervention materials
**PR and PC**	Involve local stakeholders (school governors; parents; local authorities and other agencies) to generate support for implementation.	Professional and policy stakeholder event	Included in guidance for PC.
**PR and PC**	Maintain regular contact with strategic lead at each school.	Professional and policy stakeholder event	Implemented for both PR and PC.
**PR and PC**	Highlight to schools the direct benefits to them of taking part in the trials (not just public health benefits).	Professional and policy stakeholder event	Described in manual materials for PC. Interventions mapped to school obligating to safeguard children and promote social and emotional wellbeing, and to school inspectorate judgements. For PR, confirmed inclusion of information on the impact of DRV on educational attainments in training materials.
**PR and PC**	Implement service level agreements with all schools	Professional and policy stakeholder event	SLAs implemented for PC in pilot. Timing did not work of PR.

### Consultation with students and school staff

Eight staff and nine students (five girls, four boys) from year 8 (age 12–13) participated in the Positive Choices consultations. Fifteen staff and 66 students (34 girls, 32 boys) from years 9–10 (age 13–15) participated in the Project Respect consultations (Table 2).

**Table 2:** School consultation participants

		Positive Choices	Project Respect			
			Wave 1		Wave 2^a^	
			South-east England	South-west England	South-east England	South-west England
Year 8	Girls	5	0	0	0	0
	Boys	4	0	0	0	0
Year 9	Girls	0	6	2	6	5
	Boys	0	3	4	6	6
Year 10	Girls	0	5	4	6	0
	Boys	0	6	1	6	0
**Total students**	Girls	5	11	6	12	5
	Boys	4	9	5	12	6
	**All**	**9**	**20**	**11**	**24**	**11**
**Staff**		**8**	**6**	**3**	**4**	**2**

^a^ In Project Respect, some of the wave 2 participants had also participated in wave 1

For both Positive Choices and Project Respect, staff and students generally confirmed the acceptability of intervention aims, content and modes of delivery. DRV, sexual harassment and unintended teenage pregnancy were recognised as salient issues for schools to address.

In the Positive Choices workshop, staff and students were enthusiastic about improving RSE in their school, the whole-school approach and particularly the student participatory elements of the programme. The topics suggested for coverage by the curriculum (see Table 3) also broadly mapped onto those that students wanted to be covered in year 9.

**Table 3:** Provider suggested topics for inclusion in the Positive Choices curriculum

‘Essential’ lessons	‘Add on’ lessons
1. The female/male body and functions of reproductive organs	9. Pregnancy options
2. Fertility and contraception	10. Readiness for intimacy
3. Sexually transmitted infections and safer sex	11. Body image and the digital world
4. Building blocks to good relationships	12. Female genital mutilation
5. Consent	13. Human rights, stigma and discrimination
6. Sustaining relationships	
7. Sexual response and pleasure	
8. Pornography	

The idea of tailoring the intervention to specific needs of students in each individual school was also particularly welcomed. However, some staff expressed concern about student preferences gathered from the needs survey informing curriculum topics as they felt year-8 students would not be able to accurately judge what they needed to know about relationships and sex.

Staff and students were also positive about Project Respect components. Parent engagement, a classroom curriculum, hotspot-mapping and the Circle of Six app were perceived as appropriate and deliverable  for schools. Teachers favoured the ‘train-the-trainer’ approach to staff training offered by the intervention, but highlighted that the scheduling of hour-long curriculum lessons as a potential challenge to existing timetabling. Staff suggested that there was a need for curriculum lessons to be adaptable for split delivery over shorter (usually around thirty minute) tutor-time slots or longer ‘off-timetable’ days, depending on the needs of each individual school.

With regard to intervention materials, staff in both Positive Choices and Project Respect workshops reported that, because there was so little time for implementing interventions and planning RSE outside of their academic remit, manuals needed to be comprehensive, but concise, ‘sticking to the essentials’ necessary for delivery. Similarly, teaching staff in Project Respect workshops reported a preference for ‘plug-and-play’ curriculum materials that provided detailed lesson plans, scripts to help guide classroom discussion and PowerPoint slides, so staff with limited confidence, experience or time to prepare could deliver an effective lesson.

At the same time, staff also requested some flexibility in the curriculum design to allow those with greater experience to adapt activities, including where topics had already been covered by earlier RSE provision.

In terms of the curriculum format for Project Respect, students supported proposed pedagogical approaches including the use of role-play and small-group activities particularly for discussing sensitive topics and recreating real-life scenarios. Students also agreed that it was important for the curriculum to cover less obvious forms of abuse, such as emotional abuse and controlling and coercive behaviours. They highlighted their need for training on how to respond if friends disclosed DRV as well as the importance of ensuring that lessons covered the role of social media in DRV and sexual harassment. Staff and students offered a range of terms to describe DRV and relationships, and suggested that appropriate terminology for use in the classroom should be introduced early in the curriculum. For both Project Respect and Positive Choices, students also suggested that the curricular elements on the proposed topics should be introduced before year 9, in year 7 or 8 when students are 11–13.

Students had mixed views about the acceptability of teacher-delivered RSE proposed in both interventions. Some identified benefits to delivery by staff with whom they were already familiar and had trusting relationships, suggesting this could promote better classroom discussion and reporting of safeguarding issues. However, they also associated teacher-led delivery with the risk of confidentiality breaches, and felt lessons led by teachers with whom they had less trusting or more antagonistic relationships would compromise engagement. Some students suggested that an external provider might allow more honest conversations and increase confidentiality. More important than the professional role of the educator (i.e. teacher or external provider), though, were their individual characteristics: that they were, for example, non-judgmental, able to respect confidentiality and connect with the ‘reality of young peoples’ lives’. Staff explained, however, that in practice, the selection of teaching staff would largely depend on timetabling and availability.

Across both interventions, teachers proposed that involving outside specialists could usefully cover topics they felt ill-equipped to teach, such as sexual violence and female genital cutting/mutilation. Some students and staff also felt that lessons covering more sensitive issues should be taught in single-sex groups. A suggestion was to teach some of the content in single-sex classes, but bring groups together at the end of a lesson to share learning.

### Consultation with the ALPHA group

A total of 12 young men and 10 young women participated across three ALPHA consultations (Table 4).

**Table 4:** ALPHA participants

Age in years	Positive Choices		Project Respect	
	Girls	Boys	Girls	Boys
14	2	1	0	0
15	3	2	0	1
16	1	1	0	0
17	1	0	2	2
18	1	3	0	1
19	0	1	0	0
**Total**	8	8	2	4

For Positive Choices, ALPHA members were generally supportive of the student-led social marketing element of the intervention as complementary to more formal RSE lessons on the grounds that student-led campaigns could ensure sexual health messaging was made relevant to young people. Participants raised the importance, however, of having mechanisms to ensure that campaigns were both genuinely student-led and that messages were consistent with the programme aims.

Participants broadly supported the parent component of Positive Choices, recognising the value of informing parents about the RSE being taught in school and involving them in supporting their children’s learning at home. Some participants, however, were more sceptical about resources (like homework assignments or newsletters) aiming to prompt discussion with parents and carers and felt that many students would avoid carrying out homework activities due to the risk of embarrassment or breaching existing child/parent boundaries. They also highlighted the need for flexibility in modes of engaging with parents depending on existing school practices and procedures.

For Project Respect, ALPHA consultations generally supported the use of small group and scenario-based learning activities that enabled students to reflect on ‘real-life’ scenarios. ALPHA also raised some concerns about the sensitivity of some of the Project Respect topics  and the importance of ensuring appropriate support for students who have experienced or witnessed DRV or other abuse. They suggested that, across lessons, attention to the use of online and social media in the conduct of young people’s relationships was important and should be improved.

### Consultation with practitioners and policy-makers

Twenty-three practitioner and policy-maker stakeholders from governmental and non-governmental organisations in the field of education and health attended the event.

Stakeholders were generally positive about both interventions, their theoretical basis and the whole school approach, although some were concerned that the curriculum only covered year 9 (and 10 in the case of Project Respect) rather than including a comprehensive, spiral curriculum spanning all years. They were also concerned about how the intervention might affect existing provision in schools, especially where this was already good. Participants anticipated that one of the major challenges to implementation would be ensuring schools prioritised the interventions given other pressures, and they made suggestions to address this. These included increasing engagement with head teachers and/or senior leadership teams; dissemination of programme information to all school staff; seeking ‘buy-in’ from school governors and parents; investing local partners with long-standing relationships with schools and interests in address adolescent sexual health and DRV, such as those in public-health departments, local youth organisations or school networks; and maintaining regular contact throughout implementation with a named strategic lead with enough seniority to drive action.

To facilitate school commitment, participants recommended that researchers should also highlight what schools stood to gain from the interventions beyond the improved sexual health and wellbeing of their students. This included free staff training to support continued professional development; specialist-designed curriculum materials; improved safeguarding procedures; meeting statutory obligations to support students’ social and emotional wellbeing; contribution to meeting national school-inspectorate criteria; and the potential for greater school engagement, improved pupil attendance and attainment via participatory activities and social and emotional learning. Stakeholders also suggested implementing service-level agreements with schools, although not enforceable, highlighting expectations for intervention providers, schools and researchers.

### Incorporation of feedback into intervention design

Table 1 summarises how student, staff, ALPHA and policy and practitioner feedback was incorporated into Positive Choices and Project Respect designs. Due to the timeline for the two projects with Project Respect being implemented ahead of Positive Choices, many of the findings from the Project Respect consultations could inform both interventions. The need to prioritise tight implementation timelines meant that the joint stakeholder meeting fell later than initially anticipated, and it was not possible for findings from this meeting to be fully incorporated into Project Respect prior to the start of piloting. Findings from this event nevertheless did inform the design of Positive Choices and will inform any further refinements to Project Respect.

Feedback from all stakeholders in general confirmed the acceptability of aims, components and content in both interventions, so these were not modified in preparation for formal feasibility testing. Staff concern with students having too much control over curriculum topics in Positive Choices confirmed plans to include both a set of 'essential' topics that had to be covered by all schools alongside a set of 'add on' topics that could be selected based on the student needs data (see Table 3).

Based on findings from teachers, an element of flexibility was built into both interventions, to enable the delivery of curriculum lessons in shorter periods. However, SEF (the Positive Choices specialist intervention provider) advised against delivery through single ‘off timetable’ (or ‘drop-down’) days as these can easily be missed by students and do not allow space for the accumulation, reflection and embedding of learning over time.

Manual materials were developed with teacher preferences for brevity in mind, and detailed lesson plans, slides and guidance notes were prepared for the curriculum elements of both interventions. Based on teacher feedback, some flexibility was also built into lesson plans through the incorporation of additional optional material that teachers could draw on to extend learning beyond essential items. Decisions to omit any part of the curriculums where similar provision already existed were to be managed between individual schools and the specialist provider on a case by case basis.

Based on student feedback we opted to continue with teacher delivered curriculum in both interventions, but with clear instruction on the selection criteria for teachers to deliver lessons. Suggestions to cover subtler, less obvious forms of violence and include training on how to help someone experiencing DRV confirmed planned approaches in Project Respect, while the inclusion of accurate signposting information and increased acknowledgement of the relevance of online and social media in the conduct of young people’s relationships informed further development in both interventions. The terminology identified by young people around relationships and DRV helped to define appropriate language to be used in Project Respect lessons.

Although some students had suggested that curriculum topics should be introduced earlier and professional and policy stakeholders holders had identified the need for a an age-approprtiate spiral curriculum spanning all years, this could not be incorporated into either intervention. This was because it contradicted earlier consultation in the initial proposal development phase as well as the view of the intervention providers that years 9 and 10 were the most appropriate for curriculum delivery in terms of the specific content and intensity of the respective programmes, and this had already become established in our agreed study designs and protocols. Similarly, we were unable to offer an option for external educators to compliment the curriculum elements due to budget constraints. Despite both staff and student feedback, single-sex teaching in co-educational settings was also generally not recommended so as not to limit opportunities to learn and challenge norms and values through discussion between students of different genders. Preferences to deliver in single-sex classes because of cultural or religious sensitivities were, however, to be discussed with individual schools on a case-by-case basis.

Based on student and ALPHA feedback, flexibility was built in to how the parent materials could be disseminated by schools. Homework activities in Positive Choices were also chosen to reflect ALPHA concerns that these could be embarrassing for parents and children. Activities aimed to ease into discussions at home, focussing initially on the universal, relatively less sensitive topic of ‘rites of passage’ progressing to focus on ‘abusive and healthy relationships’ in a later assignment.

ALPHA feedback regarding genuine student participation and a need for accountability of student-led marketing campaigns led to plans for the joint staff-student School Health Promotion Councils (SHPCs) to oversee student-led social marketing activity.

Strategies for increasing school engagement suggested by the professional and policy stakeholders were incorporated in to the Positive Choices manual and school communication materials, and additional school meetings and service level agreements were planned for pilot schools.

## Discussion

### Summary of key findings

Involving teachers, young people and professional and policy stakeholders in the co-production of Positive Choices and Project Respect provided valuable insights to both confirm and maximise their applicability and feasibility for implementation in English secondary schools. Consultation with schools, ALPHA and practitioner and policy stakeholders generally supported intervention aims, components, content and models of delivery. Contrary to much of the existing literature [74], students confirmed the potential acceptability of teacher-led delivery, but emphasised the need for careful selection of teaching staff, reflecting a persistent concern in the teaching of RSE in England [75, 76]. The aim of identifying, training and supporting willing and committed teachers to provide good quality curriculum delivery was, therefore, embedded within the guidance for both interventions, although, as suggested by teachers, we recognise this may not always be realisable in practice. Students and ALPHA sensitised us to the need to ensure content and materials reflected the reality of young people’s lives particularly in relation to digital culture—echoing concerns in much of the RSE literature [46, 75–77]. Students also confirmed the need for broad coverage of different types of DRV, accurate sign posting and training in supporting someone experiencing DRV and to define DRV terms clearly early on in curriculum materials.

Consultation with school staff, practitioners and policy-makers highlighted the competing priorities for school leaders’ and teachers’ time and their shrinking capacity to commit to implementing public health interventions and provided useful strategies for promoting school commitment and reducing burden on staff. Such challenges have similarly been identified elsewhere in the literature on the implementation of school-based health interventions, particularly in relation to curriculum delivery [20, 78–80]. Teacher and stakeholder feedback prompted us to develop clear and concise intervention guides and prescriptive curriculum materials in line with what stakeholders felt was workable, and to adopt strategies suggested by practitioners and policy-makers to ensure school commitment. The need for flexibility in intervention design was also incorporated by providing options to adapt how lessons were timetabled; some of the curriculum content depending on teacher time, competence and their existing school provision; and the mode through which parents were engaged. Indeed, this need for some degree of flexibility for local adaption to be embedded within complex interventions to improve potential for implementation and effectiveness is increasingly recognised [81].

A particular strength of our approach was the inclusion of a diverse range of stakeholder groups, which ensured different participants could speak with authority and provide insight on different aspects of intervention design. Students, for example, were able to express their preferences for content and delivery, enabling us to confirm or improve the relevance and acceptability of our interventions. Teachers provided insight into the current school climate and ‘what would work’ practically in terms of implementation in these settings. ALPHA members drew on their experiences of school and their training as advisors on public health research to provide authoritative views on intervention design. Practitioners and policy-makers could advise on the broader context of the English education system, particularly in relation to securing commitment and ensuring delivery in secondary schools.

However, our findings also demonstrate that there were occasions where it was not always appropriate or possible to straightforwardly adopt the advice of students, staff or other stakeholders where their perspectives contradicted existing best practice and the logic of the interventions (in the case of single-sex teaching and use of drop-down days) or the constraints of the initial study design and budget limited inclusion of recommended changes (in the case of earlier curriculum implementation, providing a spiral curriculum across years or providing external educators to compliment teacher-led lessons).

### Limitations

While the sample for the study was quite large and varied for co-production work, it was likely subject to selection bias that may have affected its representativeness. In many cases, teachers self-selected based on their interest in the topic following an invitation from school leaders, and so may have been biased in terms of their enthusiasm for sexual health programming. Although we requested a diverse and inclusive sample of students for workshops, in some cases students who were perceived to represent the school favourably may have been selected. Personal relationships with teachers and, quite simply, which students were available on the day may also have shaped these decisions. This raises important considerations about incorporating stakeholder views that may not be representative of intended recipients. The consequence could even be equity harms where interventions are co-produced in line with the cultures and preferences of some groups at the expense of others, [82]. In our case, including a range of stakeholders some of whom had a broader perspective and expertise in delivering RSE in schools may  have helped mitigate this to some extent.

Reflecting the potential implementation challenges identified in our research, pressures on school timetables and staff time also affected the scheduling of face-to-face consultation and limited the participation of some schools. Indeed, the potential burden co-production can place on participants, who may already have very full workloads, and the need to ensure that contributors are appropriately recognised and compensated for their time and work has been widely acknowledged in the literature on co-production and must be an important consideration for any future collaborative work [40, 48, 49, 83].

Finally, while acknowledging that ‘co-production’ varies as to the authority possessed by stakeholders [28, 49], we accept that there are limits to how far we can claim our own approach fits with the traditional definition of empowering participants to take an equal or lead role in intervention development [49, 84–86]. The active involvement of specialist provider agencies in the elaboration of both interventions resembled a more collaborative approach with providers drafting the materials and researchers ensuring materials aligned with the theory of change and intended outcomes. Full discussions also took place about the incorporation of stakeholder feedback, albeit with the research team leading the work and having ultimate responsibility over decision-making as contractors and owners of any new intellectual property. With students, school staff and other youth and police stakeholders the process was more instrumental and researcher-led, resembling a more consultative approach, as opposed to creating aims, components and materials a new in collaboration with students and staff and other key stakeholders themselves.

## Conclusions and implications for further research

Multi-component, whole-school interventions targeting unintended teenage pregnancy, sexual health and dating and relationships violence that involve teacher-delivered curriculum may be suitable for implementation in English secondary provided they are made adaptable to individual school settings; limit additional burden on staff; involve carerful teacher selection; and accurately reflect the realities of young people’s lives. Following refinements made via co-production further piloting of Positive Choices and Project Respect via cluster randomised trial to formally assess feasibility for implementation in English secondary schools is warranted.

Our findings demonstrate that involving potential recipients, deliverers and other stakeholders in intervention design can provide valuable insights that are likely to reduce research waste by maximising the applicability of interventions to local settings prior to formal piloting and evaluation. Co-production may be particularly useful for developing complex interventions that, like ours, must be adaptable to varying institutional contexts. We would argue, like others, that co-production can also be particularly useful and indeed necessary in developing interventions and research that addresses needs that may change rapidly, like the context of young people’s sexual relationships [44–47, 77]. Although the challenges of co-production are rarely explored, our experience also suggests that tensions can emerge where recommendations are at odds with existing best practice or the logic of interventions, or which present practical difficulties in terms of the constraints of a trial. Having from the outset well-defined, transparent procedures outling which programm elements are fixed and which are open to modification and  for deciding how stakeholder input is to be prioritised, incorporated and recompensed from the outset is therefore essential. Careful consideration over the selection of participants to ensure representitiveness of views and experiences for in intervention design is also important.

In school research specifically, the challenges we experienced with organising data generation suggest that steps need to be taken to build flexibility into timelines for intervention design (and to encourage funders to allow this) to take account of the current pressure on school timetables. A range of consultation methods is also essential to ensure that stakeholders can contribute in other ways besides face-to-face meeting. Employing multiple methods could also help to increase representation of different views and ensure all participants feel able to voice their concerns. This could include the use of anonymous consultations with broader groups using online Delphi methods, for example [64].

Finally, while it will depend on the aim of the co-production project and where it is perceived participants can most usefully contribute, it is also important to consider the potential for the involvement of intended recipients and other stakeholders to go beyond passive consultation to have more of an active role as empowered partners in the design process than they did in the work reported here. Yet while greater depth of involvement may give greater assurances of the relevance of intervention aims, approaches and materials to intended beneficiaries and the their applicability to the local implementation setting, it will bring its own challenges in terms of stakeholder burden and how to balance power in decision making to ensure interventions are locally relevant, while maintaining the opportunity to draw on existing theory and build on evidence-based approaches.

## Data Availability

The dataset supporting the conclusions of this article are available on request.

## Abbreviations

ALPHA: Advice Leading to Public Health Advancement
DECIPHer: Centre for Development, Evaluation, Complexity and Implementation in Public Health Improvement
DRV: Dating and Relationships Violence
NCB: National Children’s Bureau
NSPCC: National Society for Prevention of Cruelty to Children
PC: Positive Choices
PR: Project Respect
RSE: Relationships and Sex Education
SHPC: School Health Promotion Council
SEF: Sex Education Forum
